# Technical and tactical performance indicators discriminating winning and losing team in elite Asian beach soccer tournament

**DOI:** 10.1371/journal.pone.0219138

**Published:** 2019-06-27

**Authors:** Rabiu Muazu Musa, Anwar P. P. Abdul Majeed, Mohamad Razali Abdullah, Ahmad Fakhri Ab. Nasir, Mohd Hasnun Arif Hassan, Mohd Azraai Mohd Razman

**Affiliations:** 1 Innovative Manufacturing, Mechatronics and Sports Laboratory, Faculty of Manufacturing Engineering, Universiti Malaysia Pahang, Pekan, Malaysia; 2 Centre for Foundation and Liberal Education, Universiti Malaysia Terengganu, Kuala Nerus, Terengganu Malaysia; 3 Faculty of Applied Social Sciences, Universiti Sultan Zainal Abidin, Kuala Terengganu, Malaysia; Newcastle University, UNITED KINGDOM

## Abstract

The present study aims to identify the essential technical and tactical performance indicators that could differentiate winning and losing performance in the Asian elite beach soccer competition. A set of 20 technical and tactical performance indicators namely; shot back-third, shot mid-third, shot front-third, pass back-third, pass mid-third, pass front-third, shot in box, shot outbox, chances created, interception, turnover, goals scored 1st period, goals scored 2nd period, goals scored 3rd period, goals scored extra time, tackling, fouls committed, complete save, incomplete save and passing error were observed during the beach soccer Asian Football Confederation tournament 2017 held in Malaysia. A total of 23 matches from 12 teams were notated using StatWatch application in real-time. Discriminant analysis (DA) of standard, backward as well stepwise modes were used to develop a model for the winning (WT) and losing team (LT) whilst Mann-Whitney U test was utilized to ascertain the differences between the WT and LT with respect to the performance indicators evaluated. The standard backward, forward and stepwise discriminates the WT and the LT with an excellent accuracy of 95.65%, 91.30% and 89.13%, respectively. The standard DA model discriminated the teams from seven performance indicators whilst both the backward and forward stepwise identified two performance indicators. The Mann-Whitney U test analysis indicated that the WT is statistically significant from the LT based on the performance indicators determined from the standard mode model of the DA. It was demonstrated that seven performance indicators namely; shot front-third, pass front-third, chances created, goals scores at the 1st period, goals scored at the 2nd period, goals scored at 3rd period were directly linked to a successful performance whilst the incomplete save by the keeper attribute towards the poor performance of the team. The present finding could serve useful to the coaches as well as performance analysts as a measure of profiling successful performance and enables team improvement with respect to the associated performance indicators.

## Introduction

Beach soccer is one of the world’s fastest growing sports, particularly since FIFA began to promote and organize it through competitive matches, courses and other initiatives [1]]. The beach soccer is played on a sandy surface with a depth of at least 40 cm deep. Like basketball and futsal, the beach soccer match is divided into three periods of 12 minute with an interval time of 3 minutes between the matches. The time is stopped when the play is interrupted; therefore, the total match duration lasts for about 36 minutes. The match breaks off when the referee deems necessary, i.e. when directing for a penalty or calls for doctor assistance. Each team consists of 10 players (five players in the ground, including the goalkeeper, and five on the bench for reserve purposes). Unlike soccer, there is no restriction on the number of substitutions, hence, continues substitutions are allowed as the game progresses in order to maintain high intensity and tempo during the match. It is worth to highlight that a referee and two lines-men officiate the match.

It was reported that due to its expeditious advancement and recognition the sport was consolidated into the FIFA organization in 2004 and the initial Beach Soccer World Cup was held on Copacabana beach Brazil in 2005, with France beating Portugal in the final [2]. Since then, a number of researchers began looking at different aspects of performance elements governing the performance of the sports intending to offer insights into the nature of the sport as well as the encompassing indicators that define its competition. A study of similarities and differences between football, beach soccer and futsal in world cup tournaments matches were carried out by the preceding researchers [3]. The authors investigated the trends on the average number of goals scored per game, the incidence at which the goals were scored in between the periods of the match play as well as the effects of scoring first goal during a match and its influence towards the outcome of the match. It was established that the average goals scored per game in futsal and football tend to decline while an increase in goals scores was observed in beach soccer. It was also noted that many of the goals were scored within the last period of the games. It was reported that the probability of winning the game from scoring the first goal was about 70% in both football and futsal whereas in beach soccer it was about 60%. Finally, the authors concluded that the variations of the performance in the investigated variables were mainly attributed to the physical, technical, tactical as well as the psychological nature of each sport and therefore, each sport is unique with respect to the specific demand of the game.

In a different study, the physiological demands as well as some technical and tactical performance of Italian beach soccer players were investigated[4]. Three official games of the Italian first division beach soccer matches were assessed to evaluate the heart rate as well as the time motion analysis with respect to standing, walking, jogging, running, sprinting coupled with the technical and tactical indicators. The results indicated that the players exhaust 52.5% of the time exercising above 85% of their maximum heart rate. On the other hand, the time motion analysis demonstrated that for about 50% of the match, the players executed a considerably low-intensity activity. Moreover, the technical and tactical analysis via notational system revealed that in about 52.8% of the attacking play, at least two players were involved and of the 42.6% of the offensive attack was carried out by a single pass. It was concluded that beach soccer game is an intermittent high-intensity sport with considerable demand for anaerobic metabolism. From the time motion analysis as well as the technical and tactical aspects, the authors inferred that the sand playing surface restricts the movements of the players and as such counter their overall intensity during running.

Match demands of beach soccer between amateur and professional competitions were analysed[2]. A total of 3 matches in the Italian championship and a friendly match played by the university’s level players were investigated by the researchers. The physiological demand of the game was evaluated via the heart rate expressed as a percentage of its maximum capacity while the physical aspects were assessed using time-motion analysis. Some technical and tactical indicators were examined using notational analysis. A significant difference was observed on the heart rate levels in the three sessions, but no difference was detected between the professional team and the university players. Likewise, no distinctions were reported between the two teams in relation to the time motion analysis as well as the technical and tactical parameters. It was concluded that lack of difference observed in the heart rate values could be associated with the technical-tactical nature during the matches in which the players rapidly switched from sprinting to standing. With regards to the time motion analysis, the authors suggested that lack of variation between the two groups might be related to the difficulty of playing on the sand that is solely unconnected to the technical qualities of the players. Individualised evaluations technique was suggested based on the notational analysis employed.

Research has demonstrated that performance analysts and club coaches utilize performance indicators to evaluate the performance of a team or an individual player in order to improve performance [5]. A considerable research, especially with the regards to the motion analysis, has been conducted in beach soccer. A few of the researchers have considered integrating some performance indicators related to the sport as previously mentioned. However, a limited or no attempts have thus far devoted in establishing normative profiling of performance indicators that could determine a winner or loser in this sport. Therefore, the current investigation aims to identify performance indicators that may discriminate between winning and losing performances. It is postulated that the present investigation will offer valuable insight to coaches and performance analysts to enable better preparation that could ensure success in elite beach soccer tournament.

## Materials and methods

Before the commencement of the analysis in this study, all the relevant stakeholders (coaches, players, managers as well as the organizing committee) were informed about the purpose of the study and a verbal agreement was obtained through the Terengganu State 2017 Beach Soccer Technical Committee (AFC2017-TSG). The present study, therefore, strictly adhered to the terms of the agreement as such no video/ pictures were taken or any player’s name disclose. It is worth noting that the present study is purely observational. Moreover, some part of the data has been made publicly available by the FIFA Beach Soccer Committee in their website https://www.beachsoccer.com/events/afc-beach-soccer-championship-2017.

### Participants

The participants of the current investigation comprise of all the teams that participated in the AFC beach soccer championship 2017. A total number 12 teams participated in the match which includes China PR, Bahrain, Afghanistan, Malaysia, Oman, Lebanon, Thailand, Japan, UAE, Iraq as well as Qatar. The performance of the teams was recorded and analysed throughout the tournament. A total number of 23 matches from the 12 teams were investigated. Details information on the analysis is highlighted in the subsequent section.

### Procedures

**Fig 1:**
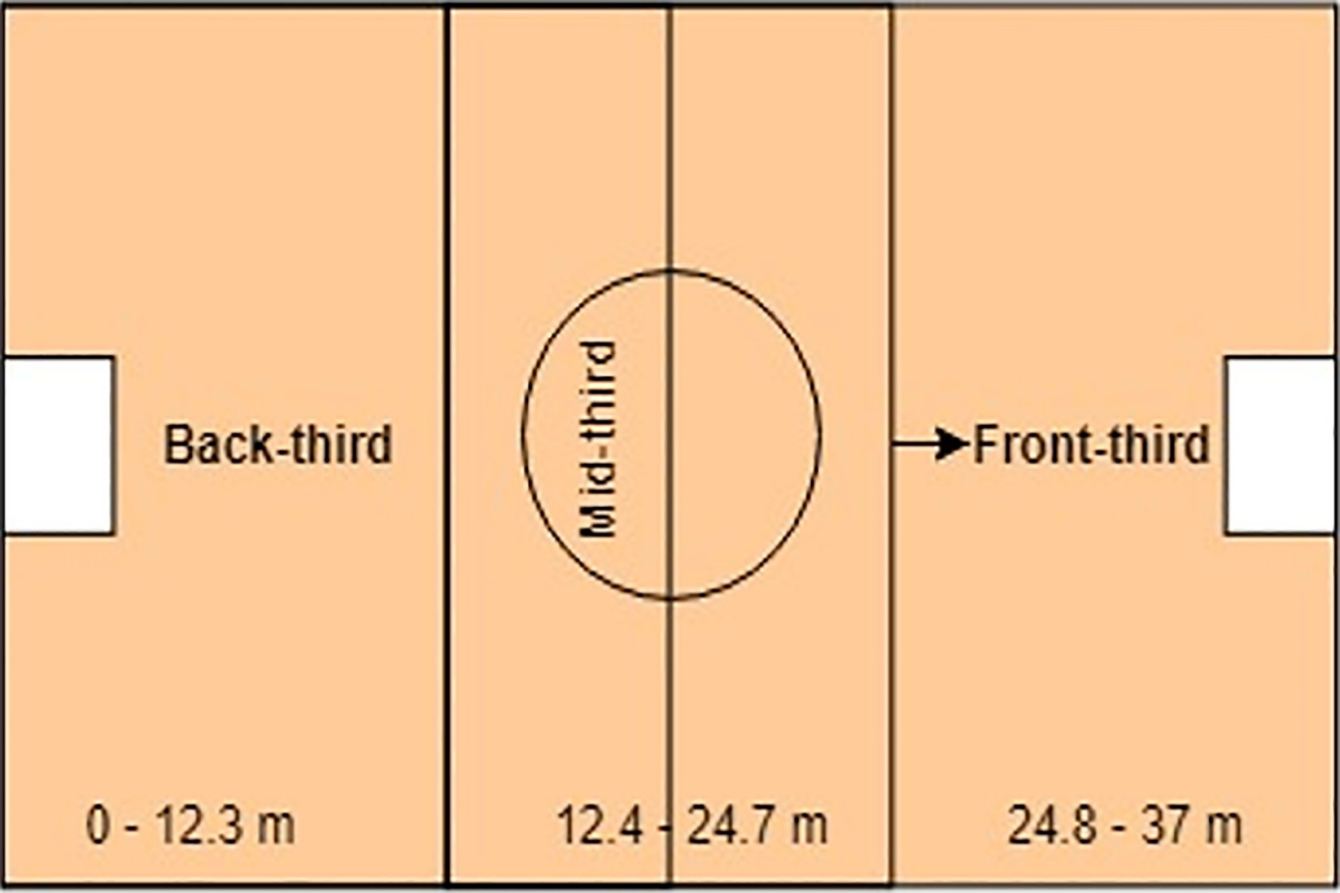
Zonal division of the pitch.

A set of 20 technical and tactical performance indicators were developed. The performance indicators namely; shot back-third, short-mid third, shot front-third, pass back-third, pass mid-third, pass front-third, shot in box, shot outbox, chances created, interception, turnover, goals scored 1st period, goals scored 2nd period, goals scored 3rd period, goals scored extra time, tackling, fouls committed, complete save, incomplete save and passing error were used to notate the performances of the teams. Four professional coaches with an average coaching experience of 10 years validated the performance indicators developed in the present study. Moreover, some of the performance indicators considered in the present study were found to be relevant to the game of beach soccer as reported in the previous studies[6,7]. The operational definitions of the performance indicators used are tabulated in Table 1. The performance indicators were considered in this investigation due to their particular relevance to the nature of the sport. The pitch was divided into three zones, i.e. back-third, mid-third and front-third where the zone in which an action occurred is taking into consideration as shown in Fig 1.

**Table 1:** Selected performance indicators and their operational definition.

Performance Indicators	Description
**Shot back-third**	Action from a player of launching the ball from the third back zone of the pitch directly to the opposing team's goal in an attempt to score
**Shot mid-third**	Action from a player of launching the ball from the mid-third zone of the pitch directly to the opposing team's goal in an attempt to score
**Shot front-third**	Action from a player of launching the ball from the front -third zone of the pitch directly to the opposing team's goal in an attempt to score a goal
**Pass back-third**	Passes successfully executed at the back-third zone of the pitch
**Pass mid-third**	Passes successfully executed at the mid-third zone of the pitch
**Pass front-third**	Passes successfully executed at the front-third zone of the pitch
**Shot in Box**	An action of directing the ball into the box of an opposing team in an attempt to score
**Shot Out Box**	An action of directing the ball outside the box of an opposing team in an attempt to score
**Chances Created**	A pass that leads to a shot on goal but not resulted in scoring a goal
**Interception**	Preventing an opponent's pass from reaching their teammates
**Turnover**	Loss of possession of a ball due to a mistake or poor control by a player
**Goals Scored 1st Period**	A successful attempt on goal that passes a goal area line during the first period of a match
**Goals Scored 2nd Period**	A successful attempt on goal that passes a goal area line during the second period of a match
**Goals Scored 3rd Period**	A successful attempt on goal that passes a goal area line during the third period of a match
**Goals Scored Extra Time**	A successful attempt on goal that passes a goal area line at the extra time of a match
**Tackling**	Dispossessing an opponent, either the tackling player gets away with the ball or not
**Fouls Committed**	An illegal attempt to dispossess a player that leads to awarding a freekick to the fouled player
**Complete Save**	A goalkeeper successfully prevents the ball from crossing the goal area line
**Incomplete Save**	The inability of a goalkeeper to prevent the ball from crossing the goal area line that leads to a goal
**Passing Error**	An error by a goalkeeper in passing a ball to a teammate

StatWatch which is a tablet based notational analysis application was utilized as a tool for data collection following the procedures previously described by the previous investigators [8]. Two independent performance analysts were recruited to notate the performance of the clubs. One performance analyst is responsible for notating the performance of a single team at a time. It is worth noting that before the commencement of the real analysis, the performance analysts were familiarized with the performance indicators developed. To establish the reliability of the analysis a different match was randomly selected. The footage of the match which contained about a total of 192 actions from all the 20 performance indicators was analysed by the experienced analysts recruited. It is worth mentioning that prior to the analysis all the performance analysts were further trained and familiarized with the choosing performance indicators such that both the analysts were using the terms unanimously. In order to establish consistency and as well as test the observational errors on the performance indicators selected, the performance analysts were instructed to notate the match individually, and their agreement was subsequently compared. A Cohen’s Kappa statistical measurement and Cronbach’s alpha analysis were utilised to assess the agreement as well as the consistency of the analysis by the performance analysts with respect to the performance indicators [9]. It is noteworthy to mention that a Kappa’s value of 0.94 and a Cronbach’s alpha of 0.98 was observed from the statistical analysis, suggesting that an excellent agreement as well as the consistency of the performance analysts in their analysis.

### Data analysis

In this study, discriminant analysis (DA) was utilized to ascertain the differences between the WT and the LT based on the performance indicators assessed. It should be noted that DA is a statistical analysis that is often employed to ascertain the variables that separate among two or more clearly joined group or classes. The DA builds a discriminant capacity for every group based on their nearness or association[10]. DA was applied in this study to determine whether the groups (winner or loser) vary with respect to the overall mean of the performance indicators assessed as well as to utilize the performance indicators in order to predict group membership. It is worth noting that the DA was selected in the present study due to its capacity to provide a wider analysis of the interactions of the assessed indicators towards the prediction of the groups i.e winner or loser from a selection of different modes. The performance indicators were used as the independent variables whilst the teams, i.e. WT and LT were used as the dependent variables. The standard, forward stepwise and backward techniques of the DA were used. It should be noted that in the standard method, the indicators are discriminated uniformly (at once) whereas, in the stepwise forward approach, the indicators are counted step by step starting with the significant indicators until no significant indicators were found. Meanwhile, in the backward stepwise method, the indicators are removed step by step beginning with the less important indicator until no more indicators are left. Moreover, the Mann-Whitney U test was employed to investigate the statistical difference between the WT and LT based on the performance indicators discriminated by DA. The statistical analysis was conducted using XLSTAT2014 add-in software for Windows. All the inferences were set at p ≤ 0.05.

## Results

Table 2 demonstrates the classification accuracies as well as the confusion matrices of the DA models. It could be seen from the table that a total of 95.65% classification accuracy was obtained from the standard mode of the DA with only two misclassifications attributed to the WT with seven performance indicators discriminated against the WT and LT. On the other hand, backward and forward stepwise models recorded a total classification accuracy of 91.30 and 89.13% with two discriminating indicators respectively. The backward stepwise mode recorded two misclassifications from both the WT and LT while the forward stepwise misclassified the two of the WT and three of the LT. It is apparent from all the models tested that standard mode models provided the best discriminating ability against the other models. The standard mode model is able to discriminate a total of seven performance indicators that potentially differentiated the WT and LT. The seven performance indicators are shot front-third, pass front-third, chances created, goals scores at the 1^st^ period, goals scored at the 2^nd^ period, goals scored at the 3^rd^ period as well as incomplete save by the keeper. These sets of performance indicators are selected for further investigation via the Mann-Whitney U test.

**Table 2:** Confusion matrix and the classification accuracy for the standard, backward stepwise and forward stepwise of the DA.

Assigned Classes	% Correct	Classification Matrix assigned by DA	
		WT	LT
**Standard mode (7 performance indicators)**			
**WT**	91.30%	2	0
**LT**	100.00%	21	23
**Total**	95.65%	23	23
**Backward stepwise (2 performance indicators) **			
**WT**	91.30%	2	2
**LT**	91.30%	21	21
**Total**	91.30%	23	23
**Forward stepwise (2 performance indicators) **			
**WT**	91.30%	2	20
**LT**	86.96%	21	3
**Total**	89.13%	23	23

Table 3 projects the Mann-Whitney U test analysis carried out on the indicators identified via the standard mode of the DA model. It could be observed from the table the all the seven performance indicators are statistically significant p < 0.01. It is, therefore, evident from the Mann-Whitney U test that the seven performance indicators mentioned above could discriminate the WT against LT in the Asian elite beach soccer tournament.

**Table 3:** Mann-Whitney U test analysis of the indicators discriminated by the standard mode DA based model.

	WT		LT		
Performance Indicators	Mean	Std Dev ±	Mean	Std Dev ±	P value (MWU Test)
**Shot front-third**	13.61	5.48	7.17	3.86	0.000
**Pass front-third**	18.78	7.82	9.83	7.19	0.001
**Chances created**	15.61	5.22	10.22	4.17	0.001
**Goals S. 1st Period**	2.26	1.57	0.65	0.83	0.000
**Goals S. 2nd Period**	2.13	1.63	0.61	0.72	0.000
**Goals S. 3rd Period**	1.87	1.29	0.87	1.01	0.008
**Incomplete save**	2.26	1.79	6.87	3.08	0.000

**Fig 2:**
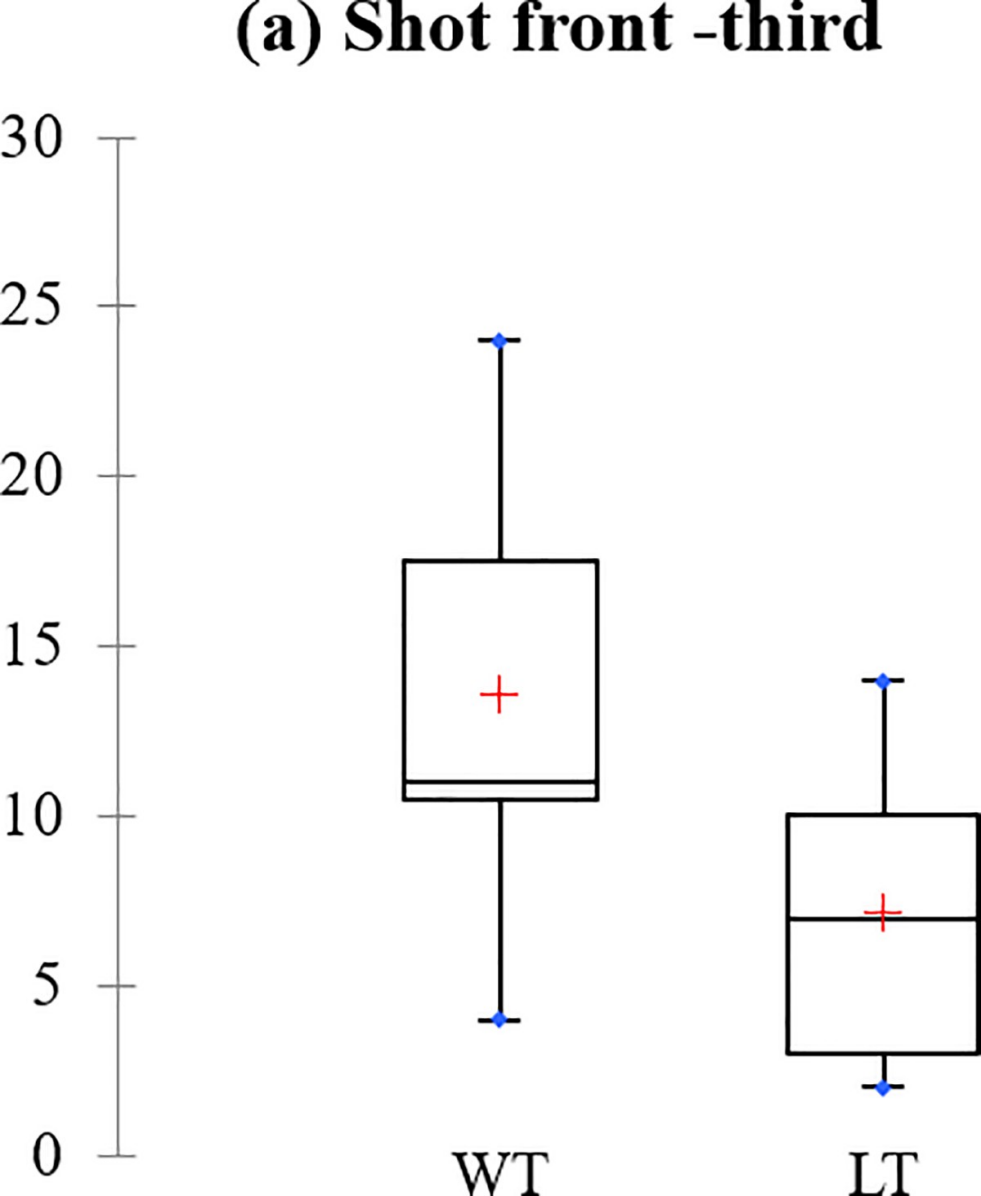
Performance differences between the WT and LT based on the standard mode model of the DA: (a) short front-third, (b) pass front-third, (c) chances created, (d) goals scored 1st period, (e) goals scored 2nd period, (f) goals scored 3rd period and (g) incomplete saves.

The boxplots in Fig 2 highlights the performance differences between the WT and LT with respect to the performance indicators discriminated by the standard mode of the DA model. It should be noted that the boxplots analysis is often used to measure the mean diffrences between two or more groups. If the mean diffrence of one group is higher than the other, it signifies that the group is superior than the other with regards to the variables measured. It is evident from the figure that the mean performances of the WT are higher in all the examined indicators except incomplete saves whereby the LT has a higher mean score. This indicated that the WT completed more shots and passed at front-third, created more chances, scored more goals at 1st, 2nd as well as in the 3rd periods and the keeper performed fewer incomplete saves as opposed to the LT.

## Discussion

The current study is the first to investigate the technical and tactical performance indicators in the Asian elite beach soccer. The study has exclusively focused on providing data to initialise the normative profile of successful performance across Asian elite beach soccer. The present study also suggests the key performance indicators that distinguish between the winning and losing team. Beach soccer is a sport that is proliferating and has been reportedly considered as one of the spectacular and exciting sport on the globe. Although many attempts have been made to explore and understand the nature of the sport in view of providing insights into the elements that influence the performance of the sports, such studies have mainly focused on physiological aspects as well as injury occurrences in the sports[1,3,11,12]. A few studies have investigated some physical and technical variables with regard to the sport [13,14]. However, no attempts were thus far made to establish the normative profile of the performance indicators that could discriminate against winning and losing teams.

The present study has demonstrated that performance indicators namely; shot at front third, pass at front third, chances created, goals scored 1st period, goals scored 2nd period, goals scored 3rd period, as well as incomplete saves, could potentially influence the chances of winning or losing in the Asian elite beach soccer. The game of beach soccer is five-a-side format which requires the players to possess a high degree of tactical intelligence. Each of the outfield players is needed to adapt to switching from both defending and attacking position. It is, therefore, essential for the players to have the mentality of shooting and to pass the ball especially at the front- third which often served as a zone where most attacks are needed to be converted into a goal[15]. Passing and shooting at the front-third zone of the pitch could provide a team with the edge in setting their attacking moves. The effectiveness of the team in passing and shooting is non-trivial to determining the success of the team.

Beach soccer is a continues-paced sport which is related in some ways with soccer. Although the beach soccer differs based on the playing surface, i.e. sand and is played using a barefoot, the game is characterised by a consistent bounce as well as the unpredictable flight of the ball. This nature of the sport reflects that many chances on goal are created. Previous researchers have inferred that chances created could be attributed to the success of a team. They opined that the more chances a team create, the higher the probability that the team might succeed in scoring a goal[16,17].

It is demonstrated in the present study that goals scored in the 1st period, 2nd period, as well as 3rd period, could determine the loser or winner in a beach soccer game. The findings indicate that the beach soccer sport is a highly demanding game both technically and tactically. Many goals are scored within a short period which necessitates a team to aim for goals irrespective of the period of the match play. This finding is congruent to the findings of the previous researchers who investigated the trends on the average number of goals scored per game as well as the incidence at which the goals were scored in between the periods of the match played between football, futsal and beach soccer. It was established from the study that the average goals scored per game in futsal and football tends to decline whilst an increase in goals scores was observed in beach soccer [13].

The goal keeper’s role in the game of beach soccer is non-trivial. The goalkeeper sets the tempo as well as the pace of the game during both the attacking and defending phases. A goalkeeper is, therefore, required to guard his goal area line securely during these phases. The inability of a goalkeeper to safeguard the goal net could result in more goals conceived since shots can be directed to the goal area line from any zone of the pitch. Similarly, a goalkeeper could be punished from a direct free kick awarded to the opposition team. Incompletes saves, or inability of the goalkeeper to stop the shots from entering the back of the net could result in losing a game [1,18].

## Conclusions

The present study has established the relevant performance indicators that could be considered as key in determining the outcome of a match in the elite Asian beach soccer competition. The performance indicators namely; shot at front third, pass at front third, chances created, goals scored 1st period, goals scored 2nd period, goals scored 3rd period as well as incomplete saves, are able to provide normative profiling that distinguishes against a winning and losing team in the sport. The aforementioned performance indicators identified could assist the coaches in mapping out strategies that could ensure winning in a game with regards to the performance indicators. This finding could also be used by the club coaches in evaluating team performance as well as targeting improvement in relation to successful performance. Moreover, the present investigation highlights a number of research avenues that are available to be carried out further in this domain, including the duplication of this study in other level of beach soccer tournament as well as extending this research into a long season beach soccer competition.

## Supporting information

S1 Table Raw data. (XLSX)
